# Waste tea as absorbent for removal of heavy metal present in contaminated water

**DOI:** 10.1016/j.heliyon.2024.e39519

**Published:** 2024-10-18

**Authors:** Saddam Husain Dhobi, Damodar Neupane, Sudhan Koirala, Deependra Das Mulmi

**Affiliations:** a Physical Science Unit, Nepal Academy of Science and Technology, Khumaltar, Lalitpur, Nepal; b Department of Physics, Patan Multiple Campus, Tribhuvan University, Patandhoka, Lalitpur, Nepal

**Keywords:** Tea, Absorbents, Heavy metals, Contaminated water, Physical and chemical properties, Removal efficiency

## Abstract

The goal of this study is to investigate the efficacy of tea-derived absorbents for the removal of heavy metals from contaminated water. For this, absorbents were prepared from milk tea (MT) waste, Ilam tea (IT) waste, and various types of tea leaves, including Immature (IML), mature (ML), and old leaves (OL). The characterization of the absorbents revealed distinct physical and chemical properties from X-ray diffraction (XRD) and Fourier transform infrared spectroscopy (FTIR), with varying degrees of effectiveness observed across different absorbent types. Heavy metal removal efficiency was assessed using a chemical-free UV-spectrophotometer. The results show that the maximum removal efficiency for Cd using the IT absorbent reaches up to 82.14 % with 100 ppm cetyltrim ethylammonium bromide (CTAB), while the minimum removal efficiency is 32.14 % using the OL absorbent with 20 ppm CTAB. For Zn, the OL absorbent demonstrates a maximum removal efficiency of 52.59 % with 100 ppm CTAB, whereas the minimum efficiency is 3.70 % using the ML absorbent with 20 ppm CTAB. For Ni, the highest removal efficiency is achieved with the ML absorbent, reaching up to 97.73 % with 100 ppm CTAB, and the lowest is 31.82 % with the MT absorbent with 20 ppm CTAB. The addition of a catalyst further enhanced removal efficiencies in both absorbents and metals. Also, the removal of heavy metal ions using 0.05 g of tea absorbent combined with CTAB at varying concentrations. Results show that the addition of 20 ppm and 100 ppm CTAB significantly reduces metal ion concentrations from an initial 100 mg/L. The metal ion concentration decreases as CTAB concentration increases, demonstrating the effectiveness of CTAB in enhancing the absorption capacity of tea absorbent for heavy metal removal. The investigation of selective adsorption of Ni, Zn, and Cd by an adsorbent, revealing competitive adsorption behavior with Ni and Zn being adsorbed more rapidly than Cd, suggesting the impact of metal ion interference on adsorption efficiency. However, limitations were observed with certain absorbent types; for instance, immature leaves did not effectively remove Ni. Overall, this study underscores the potential of tea-derived absorbents as sustainable solutions for mitigating heavy metal pollution in industrial wastewater. Further research is needed to optimize absorbent formulations and explore their applicability in real-world environmental remediation scenarios.

## 1 Introduction

Heavy metal contamination in water sources is a pressing environmental issue that poses significant risks to ecosystems and human health. Industrial activities, such as mining, manufacturing, and waste disposal, release toxic heavy metals like Cd, Ni, and Zn into water systems, leading to bioaccumulation and detrimental long-term effects. Conventional methods for removing heavy metals from water, such as chemical precipitation and ion exchange, are often costly, inefficient, and environmentally harmful. In recent years, the use of natural, low-cost adsorbents has gained attention as an alternative solution for heavy metal removal. Among these, waste products from the tea industry have emerged as promising candidates due to their abundance, affordability, and eco-friendly properties. Tea leaves contain functional groups that interact with heavy metals, making them effective in adsorption processes. However, most studies have focused on processed tea waste, with limited research on the efficacy of tea leaves at different growth stages.

Wastewater pollution due to industrialization and globalization has led to a significant increase in heavy metal levels worldwide. Tea waste, a byproduct of tea production and a widely consumed beverage, has shown the potential for removing heavy metals from wastewater. Recent research, such as that by Nandal et al. [1] and Jun et al. [2], has explored the effectiveness of using tea waste as a low-cost biosorbent for removing metals like Zn and Cu from wastewater, demonstrating promising results. Waste tea, recognized as a potential adsorbent, has been effectively utilized for removing toxic metals like Ni, Cu, Cd, and Cr from industrial wastewater [3]. Heavy metal pollution, including Cu, Cd, Cr, and Ni, remains a global environmental concern, leading to various health issues in humans [4]. Physicochemical and biological methods, such as adsorption, offer cost-effective and eco-friendly approaches for heavy metal remediation in water. These contaminants, such as Cr (VI), Cd (II), Pb (II), As (V and III), Hg (II), Ni (II), and Cu (II), pose significant threats to human health and can accumulate in the food chain and drinking water sources [5]. The presence of heavy metals interferes with metabolic pathways, causing oxidative stress and a range of adverse effects on humans and animals [6,7].

Water is indispensable for various purposes, including consumption, hygiene, agriculture, and industry. However, untreated wastewater poses a significant risk to water resources and the environment, necessitating stringent water quality standards and the removal of hazardous substances before discharge [8]. Heavy metal pollution, stemming from industrial activities like metal plating, mining, and battery manufacturing contributes to global water pollution concerns [9]. Wastewater pollution driven by industrialization and urbanization, introduces heavy metals like Cd, Pb, Cu, Zn, Ni, and Cr into the environment, posing threats to human health and ecosystems [10]. The accumulation of heavy metals in the environment and their entry into the human body through various pathways underscore the urgent need for their prevention and removal from wastewater [11]. Regulatory bodies like the World Health Organization set maximum permissible concentrations for heavy metals in drinking water to safeguard public health [12,13].

Adsorption is a cost-effective and environmentally friendly method for removing toxic metals from water. It shows high efficiency, particularly at lower metal concentrations, and the use of inexpensive adsorbents can significantly enhance removal rates [14]. Adjusting pH levels can further improve removal efficiencies, as observed with Cu(II) and Ni(II) [15]. Spent tea waste has demonstrated adsorption efficiencies ranging from 38.5 % to 100 % for lead, 11.50 %–100 % for Cd, and 100 % for mercury, making it a promising option for metal removal [16]. Heavy metal contamination in water poses significant health risks, underscoring the importance of effective removal methods [4]. Tea waste char synthesized through microwave pyrolysis has shown promise in removing hazardous metals like Cd and Hg, with removal capacities reaching 44.8 % and 50.2 %, respectively [17]. Similarly, Mahvi's investigation highlights the high efficiency of tea waste as a natural adsorbent for removing Cd, Pb, and Ni from industrial wastewater [18]. Heavy metal pollution poses a significant environmental risk, and conventional removal methods have limitations in terms of cost and effectiveness. Recent research has highlighted the potential of tea leaves and tea fibers, derived from Camellia sinensis, as low-cost, eco-friendly [19] adsorbents for removing heavy metals like cobalt, manganese, and silver from wastewater. Studies have explored adsorption mechanisms, influencing factors, and isotherms, demonstrating the effectiveness of tea-based adsorbents under various conditions [20,21]. However, no research has been conducted on the adsorption capacity of tea leaves at different developmental stages, representing a gap in current studies.

### 1.1 Statement of problem

The management of waste tea leaves generated both in tea gardens and after consumption, presents a challenge due to their random disposal. While existing literature explores the adsorption properties of commercial tea leaves in addressing heavy metal water pollution, there is a noticeable gap in research focusing on the adsorption properties of tea leaves at different stages of maturity. Specifically, the adsorption properties of OL, ML, and IML remain largely unexplored. This gap underscores the need for research to investigate how these properties vary across different stages of leaf maturity and compare them with those of commercial tea leaves. Therefore, this study aims to fill this gap by examining the adsorption properties of tea leaves at various stages of maturity using techniques such as FTIR and XRD for leaf characterization and UV-spectrophotometry for analyzing absorbance properties. By comparing the results with existing literature, a more comprehensive understanding of the adsorption properties of tea leaves can be achieved, ultimately contributing to improved waste management practices for tea leaves.

In the existing literature, tea waste has been explored as a bio-absorbent for heavy metal absorption using CTAB. However, there is a significant gap in the research as the studies have primarily focused on processed tea waste rather than exploring the absorbent potential of tea leaves at different growth stages before they are prepared as tea. The natural condition of the tea leaves and their stages (immature, mature, and old) have not been considered for their heavy metal absorption capability. This research aims to address this gap by investigating the absorbance of heavy metals using tea leaves in their natural state, without artificial synthesis or preparation. Specifically, it will compare the absorbance capacity of tea leaves at different stages of growth: immature (newborn), mature (fully harvested), and old (yellow and dried, fallen to the ground) with commercial tea waste treated with CTAB.

The novelty of this work lies in studying the absorption properties of tea leaves at different life stages, offering a fresh perspective on the natural bio-absorbent potential of tea without relying on processed waste. This investigation will help in understanding how the leaf's developmental stage influences its efficacy as a bio-absorbent, potentially paving the way for sustainable heavy metal removal methods. The objective of this research is to investigate and compare the heavy metal absorption capacity of tea leaves at different developmental stages (immature, mature, and old) in their natural state with commercially prepared tea waste treated with CTAB. The motivation behind this research is to explore a sustainable and cost-effective method for heavy metal absorption using natural tea leaves at different growth stages, without relying on artificial synthesis or chemicals. This process not only utilizes a chemical-free approach for bio-absorption but also employs an optical testing method, ensuring an eco-friendly and affordable solution for environmental remediation.

## 2 Materials and methods

### 2.1 Preparation of adsorbent

**Fig. 1:**
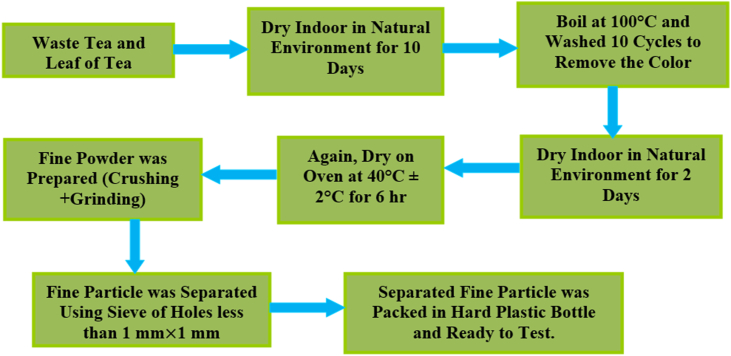
Flow diagram for preparation of absorbent particle from tea wastes.

• **MTWaste:** Tea waste from MT is initially washed several times to eliminate milk and other residues. The washed waste tea is then air-dried indoors for two days, followed by drying in a hot air oven at 40 °C ± 2 °C for 6 h. Subsequently, the dried waste tea undergoes boiling with hot distilled water at 100 °C and multiple rinses with hot water in ten cycles to remove hydrolyzable tannins, soluble components, and colorants. After this process, the sample is dried again in the oven at 40 °C ± 2 °C and crushed into a powder. The resulting powdered sample is now prepared as an absorbent for the removal of heavy metals from contaminated water.
• **ITWaste:** A similar process is undertaken to prepare the absorbent for IT waste as for ML waste. IT is one of the famous and commercial branded teas of Nepal. Both IT and MT waste absorbents are prepared in the laboratory using fresh tea obtained from the local market.
• **Leaf Tea Waste:** To prepare the absorbent from tea leaves, leaves were gathered from the tea garden, including three types: OL, ML and IML. These collected leaves were air-dried for 10 days and then subjected to oven-drying for 6 h at 40 °C ± 2 °C before being crushed into a fine powder. The powder was then boiled in hot distilled water at 100 °C to remove color, with the boiling and washing process repeated in cycles with hot distilled water for a total of 10 cycles to ensure thorough removal of color. Once the color disappeared, the solid tea material was dried indoors for 2 days and then for 6 h in the oven at 40 °C ± 2 °C. After drying, the solid material was crushed into a fine powder, resulting in the final absorbent product. All prepared samples of 5g absorbent were stored in small hard plastic bottles for testing the absorbance properties. Additionally, CTAB was stored to investigate its impact on the tea absorbent. The size of the fine powder was filtered using a sieve whose size is less than 1 mm × 1 mm and hence the fine particle size is less than 1 mm. The flow diagram of the fine particles for absorbent is shown in Fig. 1.

### 2.2 Determination of absorbance

The prepared absorbent samples derived from IT, MT, OL, MT and IML were characterized using FTIR and XRD to analyze functional groups and assess the physical properties of the absorbents. The absorbance properties of the prepared absorbents with heavy metals were investigated using a UV-spectrophotometer. For UV analysis, absorbance was measured using 3 mL of pollutant at concentrations of 20 ppm and 100 ppm CTAB. The absorbance of heavy metals is determined by the Beer−Lambert Law as shown in equation (1). The light intensity of a solution at a specific wavelength is

(1) Absorbance(A)=ε×b×c

Where ε is the absorptivity of the compound of interest at the specified wavelength, b is the path length of the cell, and c is the concentration of the test solution.

### 2.3 Preparation of pollutants

The contaminated solutions were created by dissolving heavy metal salts in deionized water, utilizing Cd, Zn, and Ni ions as model pollutants. Separate stock solutions were prepared for each metal, with concentrations of 1000 mg/L obtained using CdSO_4_.8H_2_O, Zn(NO_3_)_2_and NiCl_2_.6H_2_O, purchased from Sigma-Aldrich. Working solutions of 100 mg/L for Cd, Zn, and Ni were then prepared through serial dilutions. While natural waters typically have heavy metal concentrations up to 100 mg/L, this study demonstrated the applicability of the adsorption process in treating industrial wastewater with higher metal concentrations, as found in the battery and metal coating industries.

### 2.4 Removal efficiency of heavy metal

The concentration of the metal was determined using UV–Vis spectrophotometer at 540 nm, employing the percentage removal formula as described by equation (2) [22]:

(2) $$\text{Removal}\;\text{Efficiency}\;\left(\eta \%\right)=\frac{C_{0}-C_{e}}{C_{0}}\times 100\%$$

Where η represents the percentage of removal efficiency of the metal ion, C_0_ denotes the initial concentration of the metal ion (ppm), and C_e_ signifies the final concentration of the metal ion (ppm) after adding the absorbent. In the present study, C_e_ corresponds to the concentration after the addition of CTAB at 20 ppm and 100 ppm following 30 min of sonication. The prepared pollutant was combined with 0.05 g of absorbents (IT, OL, MT, IML, and ML) for examination. The mixture of pollutant and absorbent underwent sonication for 30 min, followed by a 5-min rest period, before absorbance measurements were taken using 20 ppm CTAB and 100 ppm CTAB. The reading was taken to study the removal efficiency of heavy metal from contaminated water prepared in the laboratory. The finding was analyzed in the result and discussion section in a tabulated and graphical way.

## 3 Results and discussion

### 3.1 FTIR spectroscopy of tea absorbents

**Fig. 2:**
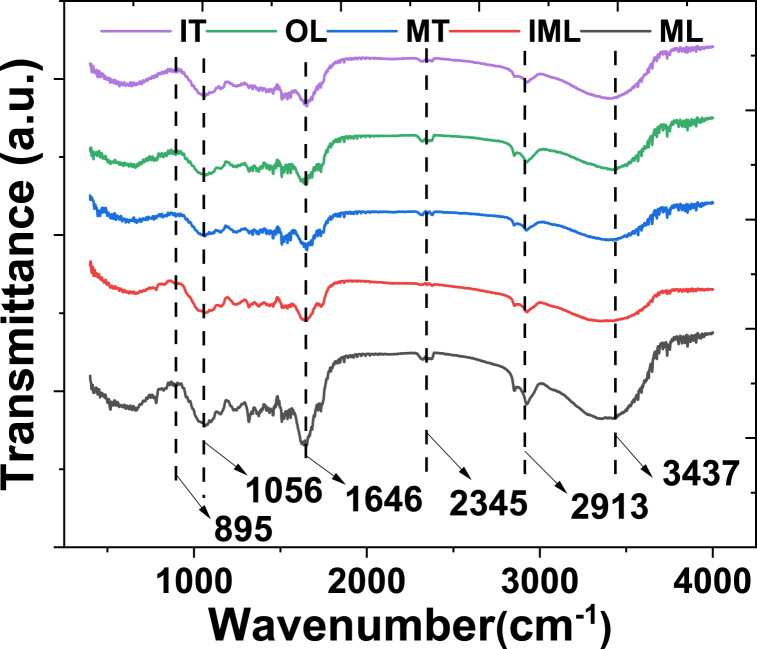
FTIR of different prepared absorbent from waste tea.

The prepared absorbent, derived from tea waste, exhibited a stem structure primarily composed of cellulose and hemicellulose. This structure displays a heterogeneous and porous surface, which is advantageous for heavy metal adsorption due to its increased surface area and distributed pores. The effectiveness of adsorption is influenced by surface roughness and pore distribution, with smaller particle sizes resulting in greater surface areas available for adsorption. The adsorption process appears to be predominantly limited to the outer surface of the absorbent, influenced by both functional groups and particle size. The main functional groups identified in the prepared absorbent, including aliphatic C–H, C–O–H, and C–O stretching functional groups, were found to be involved in adsorption, as determined by FTIR analysis as shown in Fig. 2.

We observed the presence of functional groups such as C-H, C=O, and N-H in the samples using the FTIR spectra. The transmittance order, from the highest to the lowest, was found to be in order IT > OL > MT > IML > ML of prepared absorbents, indicating differences in the concentration of functional groups among the samples, as shown in Table 1. Specifically, higher concentrations of functional groups were correlated with lower transmittance at specific wavenumbers. The composition of tea leaves, rich in cell wall components like cellulose, hemicellulose, lignin, proteins, and condensed tannins, provides hydroxyl and carboxylate functional groups capable of effectively removing contaminants. The analysis revealed distinctive functional groups present in the absorbents derived from waste tea, with notable similarities to previous studies, as shown in Table 1. In our investigation, infrared spectroscopy identified characteristic peaks corresponding to various functional groups, including S=O, -C-C-, C-Cl at 895 cm^−1^, C=O at 1646 cm^−1^, amine groups at 2345 cm^−1^, aliphatic C–H groups at 2913 cm^−1^, and -OH groups at 3437 cm^−1^. These findings align with prior research, notably with Ahluwalia and Goyal [23] for S=O, -C-C-, C-Cl, for aliphatic C–H groups, and Ebrahimian and Saberikhah [24] for -OH groups. The similarity in functional group identification supports the notion of shared chemical compositions and adsorption mechanisms across different absorbent materials, thereby affirming the effectiveness of waste tea-derived absorbents in heavy metal removal, as suggested by previous literature.

**Table 1:** Summary of function group of IT, MT, OL, ML and IML.

Wavenumber (cm^−1^) Present work	Functional group	Wavenumber (cm^−1^)	References
**895**	S=O, -C-C-, C-Cl	750–1000	[23]
**1646**	C=O	1651	
**2345**	Amine Group	2359–2342	
**2913**	Aliphatic C–H group	2916.62	
**3437**	-OH	3443	[24]

The presence of these functional groups is significant, showing the adsorption properties of tea absorbents, particularly carboxyl groups and amino acids’ functionality. These groups interact with metal ions to form ligands, facilitating the removal of contaminants such as Cu, Pb, Cr, Zn, and Se. The comparability of functional groups across different absorbent materials suggests shared mechanisms of metal adsorption. Specifically, interactions between carboxylic acid groups and carbon aromatic structures of metals on the adsorbent surface play a pivotal role in metal ion removal. Additionally, the presence of carboxylic acids, amines, alcoholic aldehydes, and amide groups enhances the adsorption of Cu and Se ions from wastewater. These results underscore the importance of both textural properties (microporosity and surface area) and heterogeneous functional groups in the adsorption capacity of absorbents for heavy metal removal, as elucidated by Vardhan et al. [25].

### 3.2 XRD analysis

**Fig. 3:**
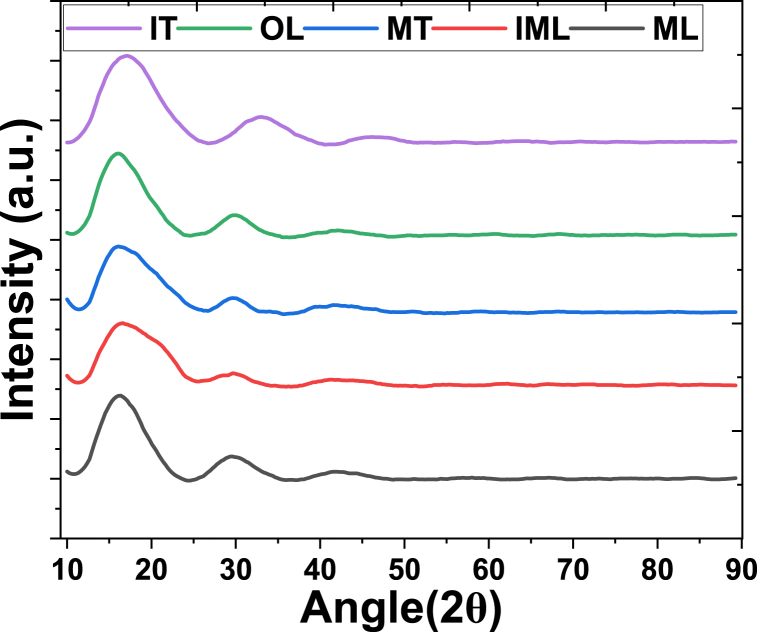
XRD of prepared absorbent from waste tea.

Fig. 3 displays the XRD patterns of the five prepared absorbent samples, revealing distinct peaks at specific angles. Two prominent peaks are evident at approximately 2θ angles of 15° and 30°, clearly distinguishable in the patterns. However, a third peak around 2θ of 42° is less distinguishable. Comparison with previous studies reveals interesting insights. Cai et al. [26] observed a similar intensity pattern in waste tea but reported a peak around 2θ of 20°, contrasting with our findings where the peak is shifted to a lower angle, approximately 15°. Furthermore, Bhoyate et al. investigated the XRD of waste tea and noted peaks around 2θ of 24° and 44° for activated tea leaves, corresponding to the (002) and (100) planes of graphitic carbon. They observed a shift towards lower angles in deactivated tea leaves, indicating increased lattice parameters upon KOH activation. Similarly, in our study, two peaks were observed, albeit at different 2θ angles compared to Bhoyate et al.'s findings, suggesting variations in lattice parameters or structural changes in the prepared absorbent samples [27]. This comparison underscores the complexity of structural transformations in waste tea-derived absorbents and highlights the importance of understanding these changes for optimizing their performance in heavy metal adsorption applications.

### 3.3 Cdmetal removal efficiency for different absorbents

**Fig. 4:**
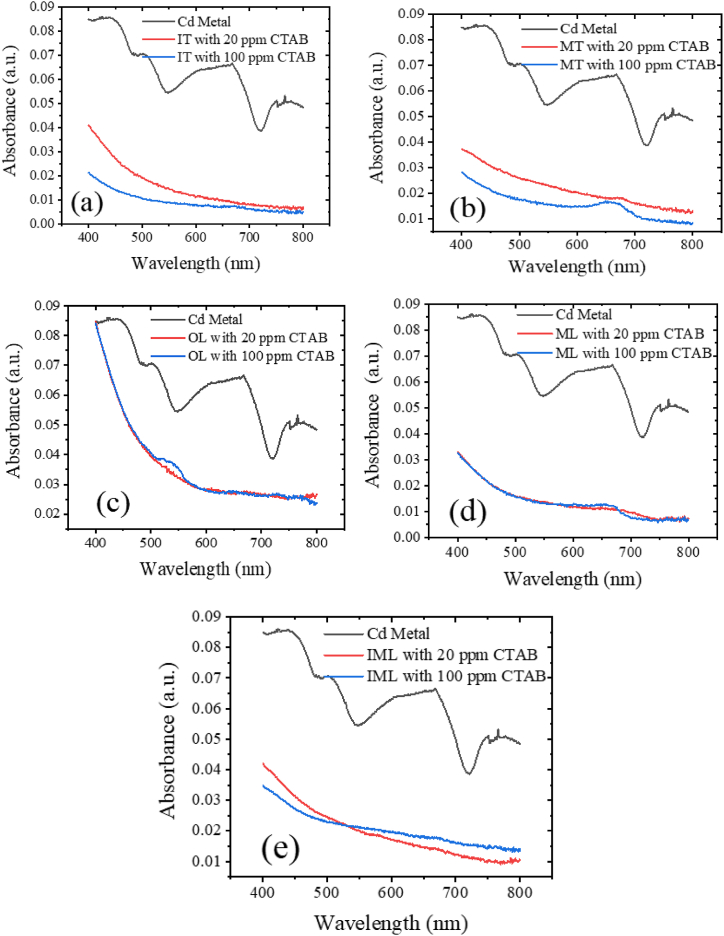
(a) IT, (b) MT, (c) OL, (d) ML and (e) IML absorbance of Cd with CTAB.

The efficiency of heavy metal removal is contingent upon the concentration of metal ions, reaching its maximum when equilibrium is achieved, after which metal ion removal ceases. Fig. 4(a)–(e) presents the absorbance of Cd heavy metal in prepared solutions when combined with tea and catalytic CTAB. The nature of absorbance Fig. 4(a) and (b) similar nature for CTAB concentration while Fig. 4(c) and (d) are different and Fig. 4(e) is many more different between Fig. 4(a) and (d). Observations indicate that with the addition of CTAB at concentrations of 20 ppm and 100 ppm, absorbance decreases, signifying an increase in transmittance, thereby indicating clearer samples due to tea's absorption of heavy metals. Table 2 illustrates the removal efficiency of heavy metals using different absorbent samples, with significant absorption of heavy metals observed with tea absorbent. Hence, utilizing waste tea for heavy metal removal proves to be a cost-effective method. Notably, this study focuses on comparing tea absorbents with leaves at three different stages: OL, ML, and IML absorbents. Comparing the absorbance nature of Cd with OL and ML absorbents reveals a similar pattern, while IML absorbent exhibits a distinct pattern. At a crossover of 20 ppm and 100 ppm around 544, IML absorbent displays an unusual nature, as the absorbance at 20 ppm CTAB approaches saturation, while at 100 ppm, it remains active for heavy metal removal. In Fig. 4(a)–(e), the lagends are only metal, which means the solution of metal is mixed with tea absorbent and free of CTAB.

**Table 2:** Summarization of removal efficiency of Cd at 545 nm.

Absorbance at 545 nm		Absorbents		Removal efficiency (%)		
ITrowhead						
0.056		Cd				
0.016		20 ppm CTAB		71.43		
0.010		100 ppm CTAB		82.14		
MTrowhead						
0.056		Cd				
0.023		20 ppm CTAB			58.93	
0.016		100 ppm CTAB			71.43	
OLrowhead						
0.056	Cd					
0.038	20 ppm CTAB		32.14			
0.015	100 ppm CTAB		73.21			
MLrowhead						
0.056	Cd					
0.013	20 ppm CTAB		76.36			
0.012	100 ppm CTAB		78.18			
IMLrowhead						
0.056	Cd					
0.025	20 ppm CTAB		55.53			
0.024	100 ppm CTAB		57.14			

Also, after adding tea absorbent 0.05 g with 20 ppm CTAB were the concentrations of heavy metal 100 mg/L decrease to 28.57 mg/L for IT, 41.07 mg/L for MT, 67.86 mg/L for OL, 23.4 mg/L for ML, and 44.47 mg/L for IML. After adding 100 ppm CTAB on 100 mg/L of heavy metal, the concentrations decrease to 17.86 mg/L for IT, 28.57 mg/L for MT, 26.78 mg/L for OL, 26.78 mg/L for ML, and 42.86 mg/L for IML. Hence, in general, the concentration of metal ion decreases with increasing CTAB. Table 2 summarizes the removal efficiency of Cd at 545 nm wavelength for various absorbent samples under different measurement conditions. For IT absorbent, the removal efficiency with 20 ppm CTAB is 71.43 %, which increases to 82.14 % with 100 ppm CTAB. MT absorbent exhibits a removal efficiency of 58.93 % with 20 ppm CTAB and 71.43 % with 100 ppm CTAB. OL shows a removal efficiency of 32.14 % with 20 ppm CTAB and 73.21 % with 100 ppm CTAB. ML demonstrates removal efficiencies of 76.36 % and 78.18 % with 20 ppm and 100 ppm CTAB, respectively. IML absorbent exhibit removal efficiencies of 55.53 % and 57.14 % with 20 ppm and 100 ppm CTAB, respectively. The observations also indicate that the removal efficiency with 20 ppm CTAB is lower compared to 100 ppm CTAB under the same experimental conditions: for the IT absorbent, it is 10.71 % lower; for the MT absorbent, it is 12.50 % lower; for the OL absorbent, notably, it is 41.07 % lower; for the ML absorbent, it is 1.82 % lower; and for the IML absorbent, it is 1.61 % lower. These results suggest that reducing the CTAB concentration from 100 ppm to 20 ppm decreases the removal efficiency for all absorbents, with the OL absorbent experiencing the most significant reduction.

### 3.4 Zn metal removal efficiency for different absorbents

**Fig. 5:**
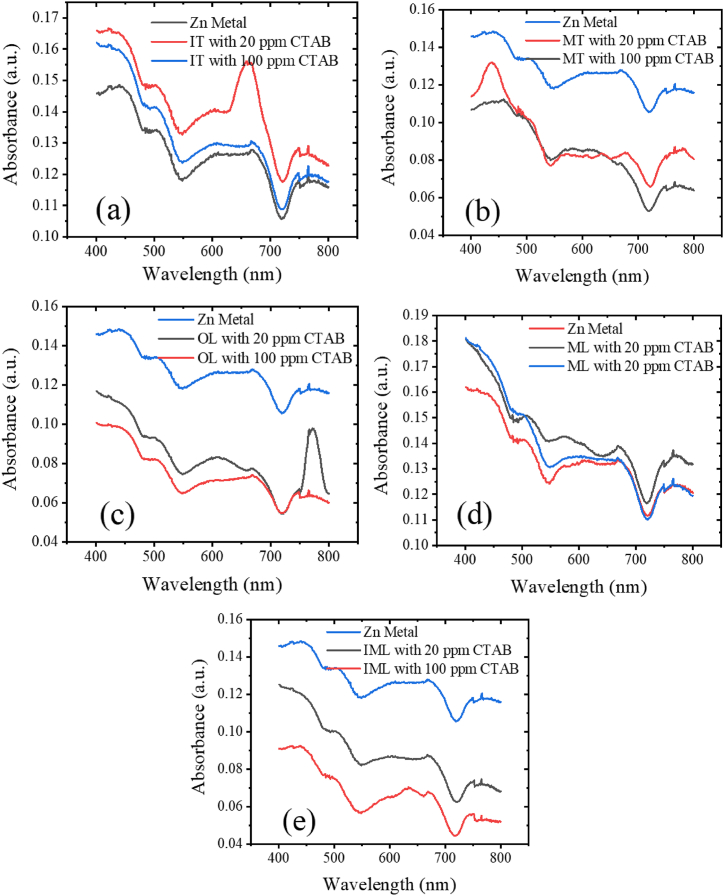
(a) IT, (b) MT, (c) OL, (d) ML and (e) IML absorption of Zn with CTAB.

The experiment conducted to investigate the absorbance of Zn heavy metal in prepared solutions mixed with tea and catalytic CTAB revealed intriguing findings. The addition of CTAB at concentrations of 20 ppm and 100 ppm resulted in a decrease in absorbance, indicating an increase in transmittance. This suggests that the addition of tea absorbent with CTAB leads to clearer samples due to enhanced absorption of heavy metals by tea. However, it was observed that absorbents prepared from IT and ML absorbents did not effectively work for Zn, as depicted in Fig. 5(a) and (d). Similarly, experiments conducted with tea without CTAB did not yield significant differences. Table 3 illustrates the removal efficiency of heavy metals using different absorbent samples. Interestingly, significant absorption of heavy metals was observed with tea absorbents derived from MT, OL, and IML absorbents, but not with IT and ML absorbents, suggesting limitations in the effectiveness of these particular samples for heavy metal removal. Further investigation is warranted to understand the reasons behind this limitation. Although numerous studies have been conducted on waste materials, this research primarily focuses on comparing tea absorbents derived from leaves at three different stages: old leaves, mature leaves, and immature leaves. Upon comparing the absorbance nature of Zn with wavelength for both OL and IML absorbents, it is observed that they are almost the same nature [Fig. 5(c) and (e)], while the absorbance of Zn with wavelength for ML absorbent exhibits a distinct pattern Fig. 5(d) and appears less effective. Additionally, it is noted that Zn with ML absorbent is effective in higher wavelength regions but not in lower wavelength regions, necessitating further investigation. These findings shed light on the complex behavior of tea-derived absorbents in heavy metal removal applications and highlight the need for tailored approaches to optimize their effectiveness.

**Table 3:** Summarization of removal efficiency of Zn at 545 nm.

Absorbance	Absorbents	Removal efficiency (%)
**IT**		
**No significant effect**	Zn	–
	20 ppm CTAB	
	100 ppm CTAB	
**MT**		
**0.135**	Zn	
**0.085**	20 ppm CTAB	37.04
**0.078**	100 ppm CTAB	42.22
**OL**		
**0.135**	Zn	
**0.080**	20 ppm CTAB	40.70
**0.064**	100 ppm CTAB	52.59
**ML**		
**0.135**	Zn	
**0.130**	20 ppm CTAB	3.70
**0.120**	100 ppm CTAB	11.11
**IML**		
**No significant effects**	Zn	–
	20 ppm CTAB	
	100 ppm CTAB	

Also, after adding tea absorbent 0.05 g with 20 ppm CTAB, the concentrations of heavy metal 100 mg/L decreased to 62.96 mg/L for MT, 59.26 mg/L for OL, and 96.29 mg/L for ML. After adding 100 ppm CTAB to 100 mg/L of heavy metal, the concentrations decrease to 57.78 mg/L for MT, 27.41 mg/L for OL, and 88.89 mg/L for ML. Hence, in general, the concentration of metal ions decreases with increasing CTAB. Table 3 presents the removal efficiency of Zn at 545 nm wavelength for various absorbent samples under different measurement conditions, focusing on the percentage of removal efficiency. For IT and IML absorbents, Zn removal efficiency was not observed under any measurement condition, indicating ineffective Zn removal by these absorbents. MT absorbent exhibited a Zn removal efficiency of 37.04 % with 20 ppm CTAB and 42.22 % with 100 ppm CTAB. OL absorbent showed the highest removal efficiencies of Zn with values of 40.70 % and 52.59 % at 20 ppm and 100 ppm CTAB, respectively. ML displayed relatively lower Zn removal efficiencies, with only 3.70 % and 11.11 % at 20 ppm and 100 ppm CTAB, respectively. The variations in Zn removal efficiency among different stages of leaf absorbents. OL absorbent demonstrated the highest removal efficiencies, followed by MT absorbent, while ML absorbent exhibited the lowest removal efficiencies. The inability of IT and IL absorbents to effectively remove Zn indicates limitations in their suitability for heavy metal removal applications. The observations also indicate that the removal efficiency with 20 ppm CTAB is lower compared to 100 ppm CTAB under the same experimental conditions: for the IT and IML absorbents, no significant results were observed; for the MT absorbent, it is 5.18 % lower; for the OL absorbent, notably, it is 11.89 % lower; and for the ML absorbent, it is 7.41 % lower. These results suggest that reducing the CTAB concentration from 100 ppm to 20 ppm decreases the removal efficiency for all absorbents, with the OL absorbent experiencing the most significant reduction.

### 3.5 Ni metal removal efficiency for different absorbents

**Fig. 6:**
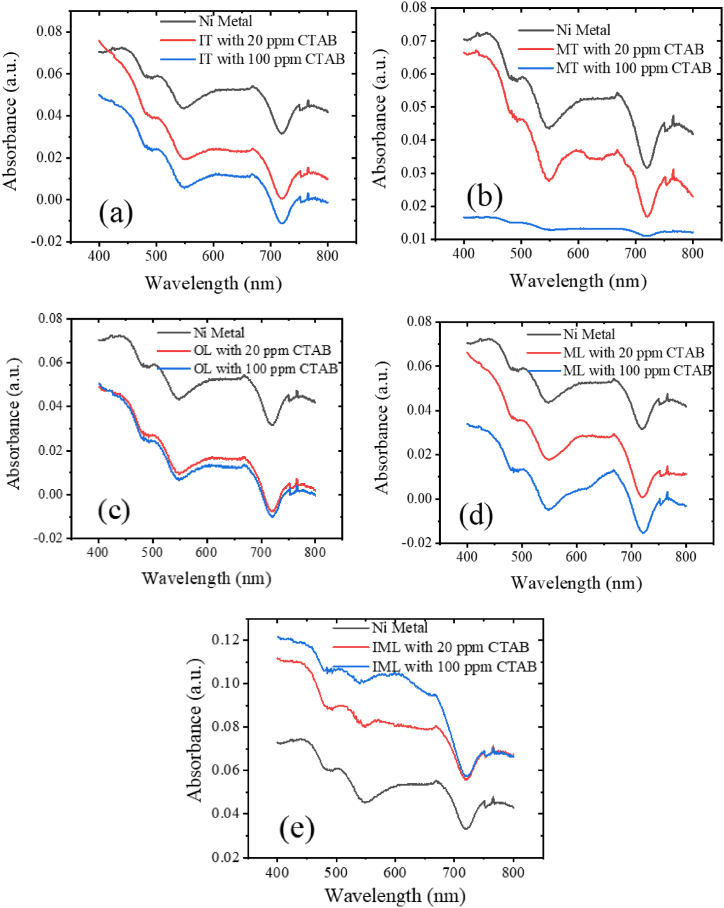
(a) IT, (b) MT, (c) OL, (d) ML and (e) IML absorbance of Ni with CTAB.

The experiment conducted, which investigated the absorbance of Ni heavy metal in prepared solutions mixed with tea and catalytic CTAB yielded significant observations. With the addition of CTAB at concentrations of 20 ppm and 100 ppm, a decrease in absorbance was observed, indicating an increase in transmittance. This suggests that the addition of tea absorbent to CTAB leads to clearer samples due to the enhanced absorption of heavy metals by tea. Fig. 6(a) and (d) has similar nature of absorbance while Fig. 6(c) is also quit similar but with CTAB concentration absorbance is almost overlapping. Fig. 6(b) and (e) are different from each other as well other figure of Ni absorbance. Similarly, experiments conducted with tea without CTAB did not yield significant differences. Table 4 illustrates the removal efficiency of heavy metals using different absorbent samples. Interestingly, significant absorption of heavy metals was observed with tea absorbents derived from IT, MT, OL, and ML, but not with IML, indicating limitations in its effectiveness for heavy metal removal. Further investigation is warranted to understand the reasons behind this limitation. Although numerous studies have been conducted on waste materials, this research primarily focuses on comparing tea absorbents derived from leaves at three different stages: old leaves, mature leaves, and immature leaves. Upon comparing the absorbance nature of Ni with OL and ML absorbents, it is observed that they almost follow the same pattern, while IML absorbent exhibits a distinct pattern and is ineffective for Ni removal.

**Table 4:** Summarization of removal efficiency of Ni at 545 nm.

Absorbance (a.u.)	Absorbents	Removal efficiency (%)
**IT**		
**0.044**	Ni	
**0.024**	20 ppm CTAB	45.45
**0.010**	100 ppm CTAB	77.27
**MT**		
**0.044**	Ni	
**0.030**	20 ppm CTAB	31.82
**0.015**	100 ppm CTAB	65.91
**OL**		
**0.044**	Ni	
**0.015**	20 ppm CTAB	65.91
**0.010**	100 ppm CTAB	77.27
**ML**		
**0.044**	Ni	
**0.025**	20 ppm CTAB	43.18
**0.001**	100 ppm CTAB	97.73
**IML**		
**No significant effect**	Ni	–
	20 ppm CTAB	
	100 ppm CTAB	

Also, after adding tea absorbent 0.5 g with 20 ppm CTAB, the concentrations of heavy metal 100 mg/L decreased to 54.55 mg/L for IT, 68.18 mg/L for MT, 67.86 mg/L for OL, and 56.82 mg/L for ML. After adding 100 ppm CTAB to 100 mg/L of heavy metal, the concentrations decrease to 22.73 mg/L for IT, 34.09 mg/L for MT, 22.73 mg/L for OL, and 2.27 mg/L for ML. Hence, in general, the concentration of metal ions decreases with increasing CTAB. Table 4 provides a concise summary of the removal efficiency of Ni at 545 nm wavelength for various absorbent samples under different measurement conditions. For IT absorbent, the removal efficiency of Ni is 45.45 % with 20 ppm CTAB and increases significantly to 77.27 % with 100 ppm CTAB. MT absorbent removal efficiency is31.82 % and 65.91 % at 20 ppm and 100 ppm CTAB, respectively. OL absorbent removal efficiencies of 65.91 % and 77.27 % at 20 ppm and 100 ppm CTAB, respectively. However, ML absorbent displays varying removal efficiencies, with 43.18 % at 20 ppm CTAB and a notably higher efficiency of 97.73 % at 100 ppm CTAB. ML absorbent shows no observed removal efficiency for Ni under any measurement conditions. This analysis underscores the effectiveness of tea-derived absorbents, particularly OL and IT absorbents, in removing Ni from solutions. The varying efficiencies across different absorbent types and CTAB concentrations highlight the importance of selecting the appropriate absorbent and catalyst for optimizing heavy metal removal processes. The observations also indicate that the removal efficiency with 20 ppm CTAB is lower compared to 100 ppm CTAB under the same experimental conditions: for the IT absorbent, it is 31.82 % lower; for the MT absorbent, it is 34.09 % lower; for the OL absorbent, it is 11.36 % lower; and notably, for the ML absorbent, it is 54.55 % lower. For the IML absorbent, no significant results were obtained. These results suggest that reducing the CTAB concentration from 100 ppm to 20 ppm decreases the removal efficiency for all absorbents, with the ML absorbent experiencing the most significant reduction.

### 3.6 Adsorption kinetics and mechanism

The rate of adsorption generally follows first-order or pseudo-first-order kinetics, where the change in the concentration of the solute is proportional to the difference between the amount of solute adsorbed at equilibrium (Q_e_) and the amount adsorbed at any time (Q_t_). The pseudo-first-order kinetic model is shown in equation (3) [28]:

(3) $$\ln\left(Q_{e}-Q_{t}\right)=\ln\;Q_{e}-\frac{k_{f}t}{2.023}$$

**Fig. 7:**
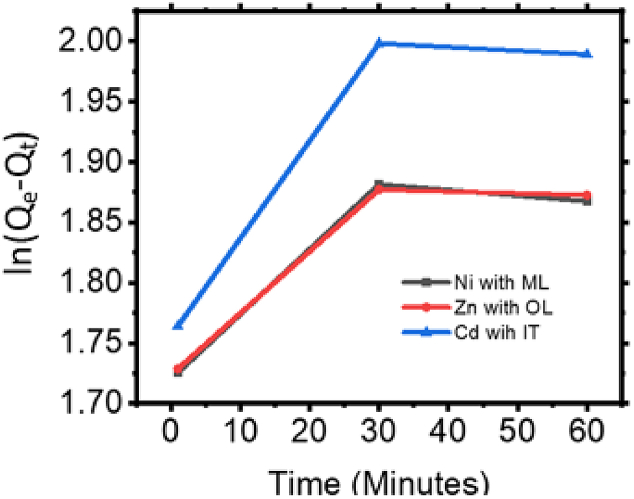
ln(Q_e_-Q_t_) with contact time of absorbent with pollution.

Here, k_f_ is the rate constant for adsorption, t is time respectively. Fig. 7 shows the nature of $\ln\left(Q_{e}-Q_{t}\right)$ with time and it is observed that at 1 minute, the values of $\ln\left(Q_{e}-Q_{t}\right)$ are lower for all metals, indicating that the adsorption process has only just started, and a significant portion of the solute is still in the solution. At 30 minutes, the values increase, suggesting that more of the solute has been adsorbed, and the system is approaching equilibrium. At 60 minutes, the values plateau, showing that the adsorption process is nearing equilibrium, with little change in the adsorbed amounts compared to 30 minutes.

The values of $\ln\left(Q_{e}-Q_{t}\right)$ for Ni increase from 1.7256 at 1 min to 1.8815 at 30 minutes but decrease slightly to 1.8675 at 60 minutes. This suggests that the adsorption for Ni is relatively fast and reaches equilibrium or a near-equilibrium state after 30 minutes. For Zn, the $\ln\left(Q_{e}-Q_{t}\right)$ values follow a similar trend as Ni, rising from 1.7287 at 1 minute to 1.8771 at 30 minutes, but with a slight decrease at 60 minutes. The slight drop indicates that the adsorption rate slowed as the system approached equilibrium. The $\ln\left(Q_{e}-Q_{t}\right)$ values for Cd show a more significant increase, from 1.7642 at 1 minute to 1.9982 at 30 minutes, indicating that Cd adsorbs slightly slower than Ni and Zn but reaches near-equilibrium by 60 minutes 1.9891. The higher values suggest that Cd has a different adsorption mechanism or binding affinity compared to the other metals.

The adsorption of these metals on the absorbent is likely following pseudo-first-order kinetics, as the values of $\ln\left(Q_{e}-Q_{t}\right)$ increase over time and approach a maximum, suggesting that the adsorption is limited by the availability of active sites on the absorbent. The differences in the values for Ni, Zn, and Cd suggest that the interaction of each metal with the absorbent surface is different. This could be due to factors larger ions may have more difficulty diffusing into the absorbent pores and different metals may have varying degrees of affinity for the adsorbent surface, depending on their charge, size, and the nature of the absorbent material. Since only 0.05 g of absorbent is added, the amount of available surface area for adsorption is limited, and the metals compete for active sites. The decrease in $\ln\left(Q_{e}-Q_{t}\right)$ values after 30 minutes for Ni and Zn suggests that most of the active sites are saturated by this time. Cd, with a higher value at 30 minutes, suggests that it may have more difficulty finding available sites or that it forms stronger bonds with the absorbent, taking longer to reach equilibrium. An observation shows that the removal of iron and magnesium with constant time increase initially and as time increase it is almost constant and decrease very slowly [29].

The adsorption of these heavy metals does not happen in isolation. In real-world applications, the presence of multiple metal ions can interfere with each other's adsorption due to competition for the same active sites on the adsorbent surface. The observation suggests that Ni and Zn are adsorbed more readily compared to Cd, which implies that Cd might be experiencing more competition from the other two metals for adsorption sites. This can be inferred from the higher values of $\ln\left(Q_{e}-Q_{t}\right)$ for Cd, indicating that even after 60 minutes, Cd has not achieved as much adsorption as Ni or Zn. Zn and Ni have relatively close $\ln\left(Q_{e}-Q_{t}\right)$ values, which suggests that their adsorption behaviors are similar. However, since Ni shows slightly higher values, it may be adsorbed preferentially over Zn when both are present in the same system. Hence, **Ni** and **Zn** are more readily adsorbed than **Cd**, indicating **selectivity** in the adsorption process. The fact that Cd is adsorbed more slowly suggests **interference** from the other two metals, likely due to competition for adsorption sites. This competitive behavior between the ions demonstrates the need to measure and understand how different metal ions influence each other's adsorption rates in multi-metal systems. Therefore, **selective adsorption** and **interference** are significant factors that must be considered when optimizing adsorbents for heavy metal removal in real-world scenarios.

The limitation of this study is that it focuses solely on three stages of tea leaves (immature, mature, and old) and compares them with two commercial teas using CTAB as a chelating agent. Additionally, the study relies on optical techniques to measure the initial and final concentrations of heavy metal ions, limiting the scope to only the selected absorbent types and analytical methods. Further exploration of other leaf stages, absorbents, and measurement techniques could provide a more comprehensive understanding of heavy metal removal efficiency.

The economic comparison between the conventional and present methods of preparing bio-absorbents reveals a compelling case for sustainability and cost-effectiveness. In the traditional approach, valuable commercial tea, priced at approximately 2.83 NRP per gram, undergoes extensive boiling and filtering, incurring significant processing and chemical costs due to the need for hazardous materials to measure heavy metal concentrations. In contrast, our innovative method utilizes waste tea leaves, which are free and sourced directly from tea gardens. This process involves simply drying and crushing the leaves, followed by a color-removal step that requires less energy and time, ultimately yielding a fine powder ready for heavy metal absorption. The present research not only eliminates material and chemical expenses but also streamlines labor, presenting a greener alternative that significantly reduces both costs and environmental impact while effectively addressing heavy metal contamination.

## 4 Conclusion

In conclusion, this study investigates the efficacy of tea-derived absorbents for the removal of heavy metals, focusing on Cd, Zn, and Ni. The experimental results demonstrate varying degrees of removal efficiency across different absorbent samples and catalytic conditions. Specifically, IT and OL absorbents exhibit promising removal efficiencies for all three heavy metals, with significant enhancements observed with the addition of CTAB catalyst. MT absorbent also shows notable removal efficiencies, albeit slightly lower compared to IT and OL absorbents. ML absorbent displays mixed results, with higher efficiencies observed for certain heavy metals at higher CTAB concentrations. However, IML absorbent demonstrates limited effectiveness for heavy metal removal. These findings underscore the potential of tea-derived absorbents, particularly IT and OL absorbents, as cost-effective and environmentally friendly solutions for heavy metal remediation in wastewater. Further research is needed to optimize the performance of tea-based absorbents and explore their potential applications in real-world environmental remediation scenarios. The experiment confirms that increasing the concentration of CTAB significantly improves the removal efficiency of heavy metals when combined with tea absorbent. Higher CTAB concentrations consistently result in lower metal ion concentrations, indicating a positive correlation between CTAB levels and heavy metal removal. Also, the adsorption of Ni and Zn occurs more efficiently than Cd, indicating selective adsorption and competition between metal ions for adsorption sites, which must be considered when optimizing adsorbents for heavy metal removal. The challenges associated with this study include determining the optimal time for maximum adsorption after the tea absorbent is added to reach equilibrium. Additionally, selecting the appropriate leaf stage from immature to old poses challenges, as does the variability in chemical concentrations of the leaves used. These factors can significantly influence the efficiency and effectiveness of heavy metal adsorption.

## CRediT authorship contribution statement

**Saddam Husain Dhobi:** Writing – review & editing, Writing – original draft, Methodology, Conceptualization. **Damodar Neupane:** Methodology, Formal analysis. **Sudhan Koirala:** Methodology, Formal analysis. **Deependra Das Mulmi:** Validation, Supervision.

## Ethical statements

This research does not conduct any experiments with living organisms.

## Conflict of interest

This work has no conflict of interest.

## Data availability statement

Data is available upon request.

## Funding sources

This work has no funding.

## Declaration of competing interest

The authors declare the following financial interests/personal relationships which may be considered as potential competing interests:Saddam Husain Dhobi and Deependra Das Mulmi reports administrative support, equipment, drugs, or supplies, and writing assistance were provided by 10.13039/501100009168Nepal Academy of Science and Technology, Khumaltar, Lalitpur, Nepal. Saddam Husain Dhobi and Deependra Das Mulmi reports a relationship with Nepal Academy of Science and Technology, Khumaltar, Lalitpur, Nepal that includes: employment. Saddam Husain Dhobi and Deependra Das Mulmi has patent pending to Not Yet. Authors declare not conflict of interest in this work. If there are other authors, they declare that they have no known competing financial interests or personal relationships that could have appeared to influence the work reported in this paper.
